# Diagnostic Accuracy and Clinical Utility of an Ayurvedic Questionnaire for Predicting Metabolic Syndrome

**DOI:** 10.7759/cureus.107562

**Published:** 2026-04-23

**Authors:** Gunjan P Bhusari, Kiran A Tawalare, Shraddha P Bawane, Priya S Meshram, Vicky Pawar, Pankaj Jogi, Kalpana Tawalare

**Affiliations:** 1 Department of Kriya Sharir (Human Physiology), Maharashtra University of Health Sciences, Nashik, IND; 2 Department of Kriya Sharir (Human Physiology), Shri Ayurved Mahavidyalaya, Nagpur, IND; 3 Department of Rachana Sharir (Anatomy), Indutai Gaikwad Patil Ayurvedic Hospital and Research Centre, Nagpur, IND; 4 Department of Rachana Sharir (Anatomy), Maharashtra University of Health Sciences, Nashik, IND

**Keywords:** diagnostic accuracy, medovaha srotas dushti, metabolic risk, metabolic syndrome, screening tool

## Abstract

Background: Metabolic syndrome (MetS) is frequently underdetected despite cardiometabolic risk. A validated 13-item *Medovaha Srotas Dushti *(MSD) questionnaire operationalises an Ayurvedic construct of lipid-metabolic dysfunction, but its clinical concordance with biomedical MetS criteria requires evaluation.

Objective: This study aimed to determine the association between MSD and MetS and to assess the diagnostic accuracy of the MSD questionnaire against National Cholesterol Education Program Adult Treatment Panel III (NCEP-ATP III) 2005 criteria.

Methods: In an 18-month cross-sectional analytical study conducted at Shri Ayurveda Hospital, Nagpur, and its peripheral outreach settings (including hospital- and community-based participants), 218 adults aged 31-60 years were screened and 90 enrolled. Eligible participants meeting the inclusion criterion (age 31-60 years) completed the MSD questionnaire (dichotomous scoring); scores ≥70% (≥9 out of 13) were classified as MSD-positive. Anthropometry, blood pressure, fasting glucose, triglycerides, and high-density lipoprotein (HDL) cholesterol were measured. Associations were tested using chi-square (χ²) and risk ratios (RRs); logistic regression adjusted for obesity grade and sex. Diagnostic indices and Cohen’s kappa (κ) were calculated.

Results: MetS was present in 53.3% of participants. MSD-positive participants showed significantly higher adiposity measures, blood pressure, triglycerides, and fasting glucose, and lower HDL (all p < 0.001). MSD was strongly associated with MetS (χ² = 64.46, p = 0.001; RR = 18.81, 95% CI 4.85-73). The questionnaire demonstrated 95.56% sensitivity, 88.89% specificity, and 92.22% overall accuracy with excellent agreement (κ = 0.844).

Conclusion: MSD was significantly associated with MetS, and the MSD questionnaire demonstrated high diagnostic agreement with NCEP-ATP III criteria. These findings suggest its potential as a non-invasive adjunct screening tool for identifying individuals at increased metabolic risk. However, results should be interpreted cautiously due to the cross-sectional design; they do not establish causality or independent predictive validity and require further validation in larger longitudinal studies.

## Introduction

Metabolic syndrome (MetS) is a significant and increasing public health problem, characterised by a cluster of central obesity, dyslipidaemia, hypertension, and impaired glucose regulation [1]. This constellation reflects underlying disruptions in lipid metabolism, insulin sensitivity, adipose tissue signalling, and inflammatory pathways, leading to an increased risk of cardiovascular disease and type 2 diabetes mellitus [2]. The global prevalence of MetS has risen with increasing trends in obesity, physical inactivity, and unhealthy dietary habits [3]. A widely accepted definition by international health organisations includes abdominal obesity, hypertriglyceridemia, reduced high-density lipoprotein (HDL) cholesterol, hypertension, and elevated fasting blood glucose levels [4]. MetS often remains undiagnosed in its early stages, as symptoms are frequently absent and cardiometabolic complications typically manifest later, making early identification a persistent challenge in clinical practice [5]. The burden of metabolic abnormalities is particularly prominent in individuals aged 31-60 years, a life stage associated with increased risk due to cumulative lifestyle and metabolic factors. Conventional screening approaches rely primarily on biochemical and anthropometric measurements, which may not always be feasible for early, large-scale, or resource-limited population screening, thereby highlighting the need for simple, non-invasive, and cost-effective screening tools.

Ayurvedic classical texts provide a functional framework for understanding metabolic imbalances through the concept of *Srotas *(channels), which are physiological pathways responsible for the transport, transformation, and nourishment of tissues [6]. Lipid metabolism and adipose tissue regulation are governed by the *Medovaha Srotas *(fat-carrying channels). *Medovaha Srotas Dushti *(MSD) (vitiation of fat-carrying channels) is described as a pathological condition arising from impaired lipid synthesis, circulation, and utilisation, leading to excess fat accumulation, disturbed glucose metabolism, and increased susceptibility to cardiovascular diseases [7]. Classical literature attributes this dysfunction to unhealthy dietary patterns and lifestyle factors such as excessive intake of energy-dense foods, alcohol consumption, sedentary behaviour, and daytime sleep [8]. These descriptions show conceptual similarity to the biomedical pathophysiology of MetS, particularly in relation to obesity-driven metabolic dysregulation. This conceptual overlap suggests that Ayurvedic functional assessment tools may capture early metabolic disturbances before overt biochemical abnormalities become evident.

To operationalise the classical conceptual framework into a clinically applicable format, a symptom-based 13-item MSD questionnaire was developed and subsequently evaluated using standard psychometric validation methods [9]. The instrument demonstrated good content validity, internal consistency, and construct validity, supporting its reliability in measuring functional lipid metabolic disturbances. However, psychometric validation alone does not establish clinical applicability. There is limited evidence regarding the extent to which MSD scores correspond to established biomedical criteria for MetS or whether the questionnaire can reliably identify individuals at increased metabolic risk. Therefore, beyond psychometric validation, it is essential to evaluate the diagnostic accuracy, agreement, and clinical utility of the questionnaire against established biomedical standards.

Evaluating the correlation between MSD and MetS is necessary to determine the clinical relevance of this validated tool. Demonstrating agreement between an Ayurvedic functional assessment and standard metabolic parameters may support its role as a complementary screening instrument within an integrative metabolic risk assessment framework. Establishing such diagnostic concordance could facilitate the incorporation of this non-invasive questionnaire into routine clinical and community-based screening approaches for identifying individuals at increased metabolic risk.

Objectives of the study

The primary objective of this study was to assess the diagnostic accuracy of the MSD questionnaire in identifying MetS using the National Cholesterol Education Program Adult Treatment Panel III (NCEP-ATP III) 2005 criteria as the reference standard [10].

The secondary objectives were to evaluate the association between MSD and MetS, assess the agreement between MSD-based classification and established diagnostic criteria, and determine the clinical utility of the questionnaire as a non-invasive screening tool for identifying individuals at increased metabolic risk.

## Materials and methods

Study design and setting

The study employed a cross-sectional analytical design and was conducted over a total duration of 18 months at Shri Ayurveda Hospital, Nagpur, and its peripheral outreach settings, including both hospital- and community-based populations. The study was conducted among adults attending outpatient departments and peripheral settings. The study population included individuals aged 31-60 years. The study setting was selected to ensure a heterogeneous sample comprising both hospital attendees and community participants, representing diverse metabolic risk profiles, thereby facilitating the assessment of the relationship between functional metabolic evaluation and established biomedical criteria for MetS.

Ethical considerations

The study was initiated following ethical clearance from the Institutional Ethics Committee (Approval No.: SAMN/IEC/MD/05/2023). All procedures were conducted in accordance with the ethical principles outlined in the Declaration of Helsinki. Participants were informed about the study objectives, procedures, potential benefits, and data confidentiality. Written informed consent was obtained from all participants prior to enrolment. Participation was voluntary, and individuals were free to withdraw at any stage without affecting their routine medical care.

Study population and sampling

A total of 218 individuals were initially screened. The study sample was selected using a quota sampling method to ensure equal representation of MSD-positive and MSD-negative participants. Participants were recruited from outpatient departments and peripheral outreach settings using a sequential screening approach. Individuals were first assessed using the MSD questionnaire and subsequently classified as MSD-positive or MSD-negative based on their scores. Predefined quotas were applied to ensure equal representation of both groups (45 participants each), and participants were selected accordingly to maintain balanced group allocation.

**Figure 1:**
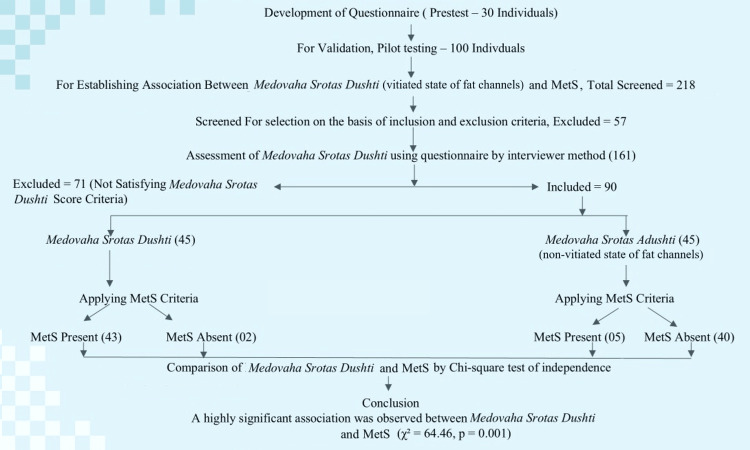
Study flow diagram of participant selection and analysis for MSD and MetS MSD: *Medovaha Srotas Dushti*; MetS: Metabolic syndrome; χ²: chi-square

The inclusion criteria comprised adults aged 31-60 years recruited from Shri Ayurveda Hospital, Nagpur, and its peripheral (community and outpatient) settings, who were able to provide informed consent. The exclusion criteria included a prior diagnosis of hypertension, diabetes mellitus, hypothyroidism, polycystic ovary syndrome, pregnancy, and lactation, as these conditions could independently influence metabolic parameters. A final sample of 90 participants was enrolled after applying the eligibility criteria. Figure 1 illustrates the participant flow and classification based on MSD status and MetS outcomes.

Sample size determination

The sample size for the analytical phase was calculated a priori based on a reported prevalence of MetS of 20%, using the standard formula: 

$n=\frac{Z^2p(1-p)}{d^2}$

Where n = sample size, Z = standard normal deviate (e.g., 1.96 for 95% confidence), p = estimated prevalence, and d = allowable error (precision).

With a 95% confidence level (Z = 1.96) and allowable precision, the estimated sample size was approximately 45 per group, resulting in a total sample size of 90 participants. The pilot testing phase, which included 100 participants, was conducted separately for psychometric validation of the questionnaire and was not included in the final analytical sample.

MSD questionnaire description and scoring

The MSD questionnaire employed in this study was previously developed and psychometrically validated by the authors [9] and was administered using an interviewer-based method, thereby eliminating the need for translation into a local language. The instrument consists of 13 symptom-based items derived from a classical literature review, expert validation, pretesting with 30 individuals, and pilot testing with 100 participants. The questionnaire demonstrated good psychometric properties, including high content validity (Scale Content Validity Index/Average (S-CVI/Ave) = 0.90), strong face validity (score: 90.77), internal consistency (Cronbach’s alpha (α) = 0.813), and satisfactory construct validity as evidenced by exploratory factor analysis. All items were scored dichotomously, with positive responses assigned 1 point and negative responses assigned 0 points.

All questionnaires were administered by trained investigators following a standardised protocol. The same trained interviewer conducted the majority of assessments, and periodic supervision was implemented to ensure consistency and minimise inter-interviewer variability. The full questionnaire is provided in Appendix A for reference.

Classification of MSD status

The total MSD questionnaire score ranged from 0 to 13. Participants scoring ≥70% (≥9 out of 13) were classified as MSD-positive, indicating functional derangement of lipid metabolism pathways, while those scoring below 70% were classified as MSD-negative. The cutoff (≥70%) was adopted from prior validation studies of the MSD questionnaire [9], where it demonstrated optimal discrimination; therefore, a predefined validated threshold was used rather than deriving a new cutoff using receiver operating characteristic (ROC) curve analysis in the present study. All participants completed the questionnaire under uniform conditions administered by trained investigators to minimise interviewer-related variability.

Anthropometric, clinical, and biochemical assessment

Standardised procedures were used to collect anthropometric measurements, including height, weight, body mass index (BMI), and waist circumference. Waist circumference was measured at the midpoint between the lower margin of the last palpable rib and the top of the iliac crest. Blood pressure was measured using a calibrated sphygmomanometer in accordance with established clinical guidelines, with participants in a seated position after adequate rest. Fasting blood samples were analysed in certified laboratories to determine fasting blood glucose, HDL cholesterol, and serum triglyceride levels. All biochemical analyses were performed under fasting conditions (8-12 hours) using standardised laboratory protocols to ensure reliability and consistency.

All laboratory investigations were conducted in a National Accreditation Board for Testing and Calibration Laboratories (NABL)-accredited laboratory using standardised assay methods. Internal quality control procedures were performed routinely, and external quality assurance was maintained through participation in proficiency testing programs. Laboratory instruments were regularly calibrated, and standard operating procedures (SOPs) were strictly followed to ensure accuracy, reliability, and reproducibility of biochemical measurements.

Definition of MetS

MetS was defined according to the National Cholesterol Education Program Adult Treatment Panel III (NCEP-ATP III) 2005 criteria [10]. A diagnosis required the presence of at least three of the following five components: elevated fasting plasma glucose, increased waist circumference, elevated serum triglycerides, reduced HDL cholesterol, and elevated blood pressure. This definition is widely accepted and used in clinical and epidemiological studies.

Statistical analyses

All statistical analyses were performed using IBM SPSS Statistics version 26.0 (IBM Corp., Armonk, USA) and STATA version 14.0 (StataCorp LLC, College Station, USA). Standard epidemiological and psychometric methods were applied. Continuous variables were expressed as mean ± standard deviation (SD), while categorical variables were presented as frequencies and percentages (n (%)). Comparisons between participants with and without MetS were performed using independent Student’s t-tests for continuous variables and chi-square (χ²) tests for categorical variables. Statistical significance was set at p < 0.05.

The association between MSD status and MetS was evaluated using risk ratios (RRs) with 95% CIs. Binary logistic regression analysis was conducted to assess whether MSD was an independent predictor of MetS after adjusting for obesity grade and sex. Obesity grade was defined based on BMI according to the WHO classification: normal weight (18.5-24.9 kg/m²), overweight (25.0-29.9 kg/m²), and obesity (≥30 kg/m²) [11]. Effect sizes were expressed as odds ratios (ORs) and RRs with corresponding 95% CIs. Diagnostic accuracy of the MSD questionnaire was evaluated using sensitivity, specificity, positive predictive value, negative predictive value, overall accuracy, and Cohen’s kappa (κ) coefficient to assess agreement with MetS diagnostic criteria. All relevant estimates were reported with 95% CIs where applicable.

## Results

Participant characteristics

The final analysis included 90 participants aged 31-60 years. Participants were categorised as MSD-positive or MSD-negative based on the validated MSD questionnaire. MetS was diagnosed according to the NCEP-ATP III criteria. MetS was identified in 48 participants (53.3%), indicating a considerable burden of metabolic risk within the study population (Table 1).

**Table 1:** Gender distribution among participants with and without MetS Values are expressed as n (%). Statistical significance was defined as p < 0.05. MetS: metabolic syndrome; χ²: chi-square

Gender	MetS Present, n (%)	MetS Absent, n (%)	χ²	p-value
Male	21 (43.75%)	20 (47.62%)	0.14	0.713
Female	27 (56.25%)	22 (52.38%)	-	-

Comparison between the MetS present and absent groups

Participants with MetS showed significantly higher anthropometric, hemodynamic, and biochemical parameters compared with those without MetS (Table 2).

**Table 2:** Comparison of clinical and biochemical parameters between the MetS present and absent groups Values are expressed as mean ± SD. An independent sample t-test was used for group comparison. A p-value < 0.05 was considered statistically significant. MSD: *Medovaha Srotas Dushti*; MetS: metabolic syndrome; SD: standard deviation; BP: blood pressure, HDL: high-density lipoprotein; BMI: body mass index

Variable	MetS Present (n = 48), mean ± SD	MetS Absent (n = 42), mean ± SD	t-value	p-value
Age (years)	47.81 ± 8.78	45.86 ± 7.89	1.10	0.272
Height (m)	1.63 ± 0.06	1.65 ± 0.07	-1.40	0.164
BMI (kg/m²)	32.06 ± 3.12	22.13 ± 2.17	17.80	<0.001
MSD score	10.35 ± 2.93	2.07 ± 2.73	14.90	<0.001
Waist circumference (cm)	105.21 ± 4.73	89.90 ± 5.43	13.70	<0.001
Systolic BP (mmHg)	150.42 ± 12.87	111.19 ± 8.02	17.60	<0.001
Diastolic BP (mmHg)	99.58 ± 5.44	81.43 ± 6.08	15.20	<0.001
Triglycerides (mg/dL)	286.08 ± 46.63	145.91 ± 8.77	17.30	<0.001
HDL (mg/dL)	39.09 ± 1.60	51.64 ± 2.97	-23.40	<0.001
Fasting blood sugar (mg/dL)	181.54 ± 40.04	87.88 ± 8.39	14.80	<0.001

Anthropometric and hemodynamic findings

The MSD-positive participants reported a high anthropometric value connected with adiposity in comparison to the MSD-negative ones. The indicators of central obesity and general body mass were always higher in the MSD-positive group. Also, systolic and diastolic blood pressure were considerably increased in subjects with MSD, indicating a more significant frequency of hemodynamic deviations typically related to metabolic dysfunction.

Biochemical profile

Biochemical assessment demonstrated a significantly unfavourable metabolic shape of MSD-positive participants. Dyslipidemic and impaired glucose regulation markers were also much more severe in this group. There were high levels of triglyceride and fasting glucose and low levels of HDL cholesterol, which represent typical insulin-resistance-related and cardiometabolic-risk characteristics. Such results support the relationship between functional lipid metabolism disorders and known biochemical MetS elements.

Association between MSD and MetS

The prevalence of MetS differed significantly according to MSD status. Individuals classified as MSD-positive showed a markedly higher proportion of MetS compared with those classified as MSD-negative. Table 3 presents the statistical strength and significance of this association.

**Table 3:** Association between MSD and MetS Values are expressed as n (%). Statistical significance was defined as p < 0.05. RR = 18.81, 95% CI: 4.85-73.0 MSD: *Medovaha Srotas Dushti*; MetS: metabolic syndrome; RR: risk ratio; χ²: chi-square

MSD Status	MetS Present, n (%)	MetS Absent, n (%)	χ²	p-value
MSD Present	43 (89.58%)	2 (4.76%)	64.46	0.001
MSD Absent	5 (10.42%)	40 (95.24%)	-	-

**Figure 2:**
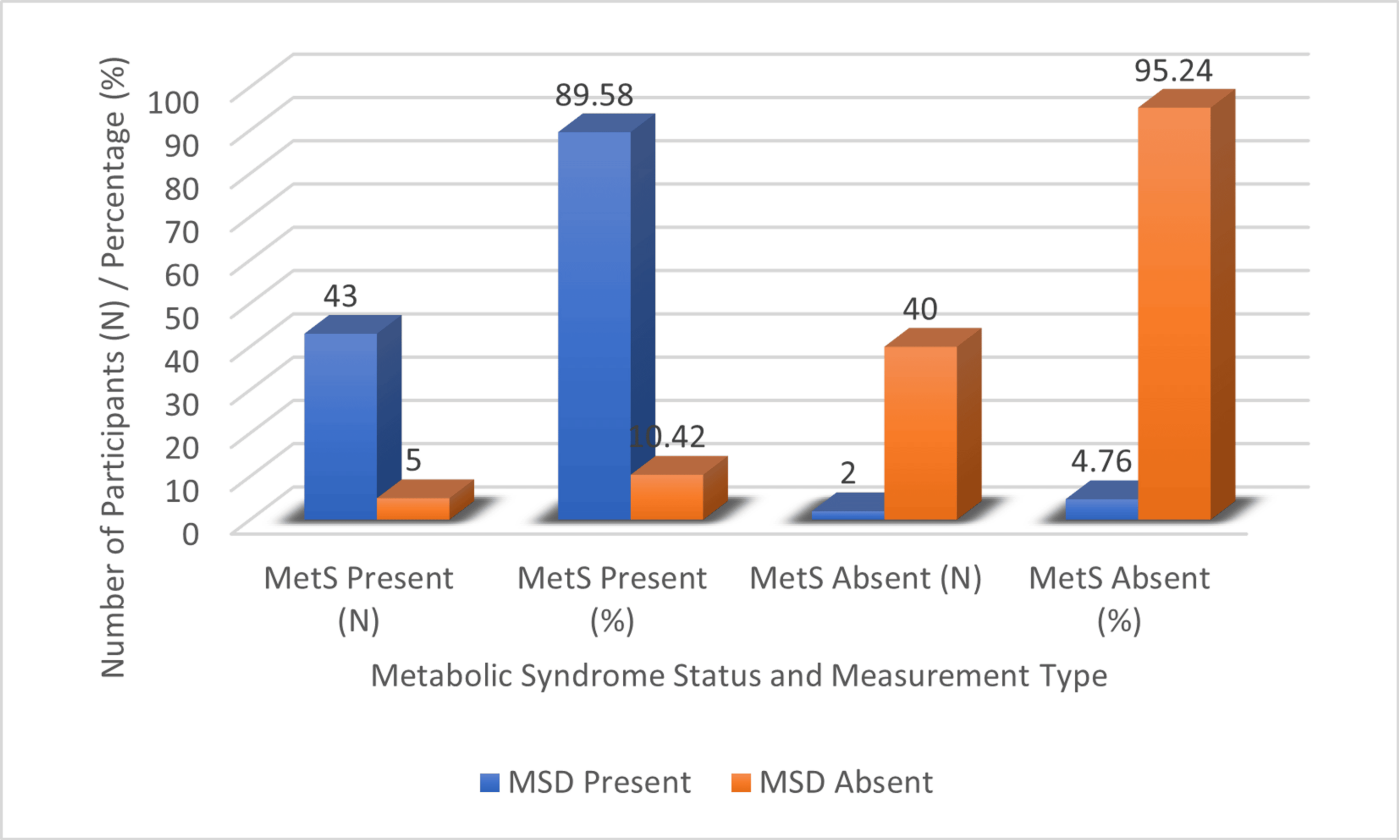
Association between MetS and MSD MSD: *Medovaha Srotas Dushti*; MetS: metabolic syndrome

The distribution pattern of MetS across MSD categories is illustrated in Figure 2.

Multivariable logistic regression analysis

Multivariable binary logistic regression analysis was performed to assess the independent association between MSD and MetS after adjusting for obesity and sex. The regression model demonstrated overall statistical significance. After adjustment, MSD was not independently associated with MetS, whereas obesity emerged as a significant independent predictor. Sex was not significantly associated with MetS in the adjusted model. The detailed results of the regression analysis are presented in Tables 4-5.

**Table 4:** Multivariable logistic regression analysis for predictors of MetS Values are expressed as n (%). Statistical significance was defined as p < 0.05. MSD: *Medovaha Srotas Dushti*; MetS: metabolic syndrome; OR: odds ratio

Variable	OR	z-value	p-value	95% CI
MSD	0.62	0.29	0.771	0.02-15.09
Obesity	72.98	3.35	0.001	5.94-896.8
Sex	2.33	0.75	0.453	0.25-21.23

**Table 5:** Model statistics LR: likelihood ratio; Prob: probability; R²: coefficient of determination; χ²: chi-square

Parameter	Value
Number of observations	90
LR χ²	97.93
Prob > χ²	0.0001
Log likelihood	-13.32
Pseudo R²	0.7858

Diagnostic performance of the MSD questionnaire

The MSD questionnaire was found to have a good internal consistency and significant diagnostic ability to identify MetS. Diagnostic validity measures showed that MSD-based classification was highly agreeable with established criteria of MetS. Reliability and diagnostic accuracy metrics are summarised in Table 6.

**Table 6:** Diagnostic validity of the MSD questionnaire Values are expressed as percentages (%) with corresponding 95% CIs where applicable. Cronbach’s α was used to assess internal consistency, and Cohen’s κ was used to evaluate agreement. A p-value < 0.05 was considered statistically significant. MSD: *Medovaha Srotas Dushti*

Parameter	Value	95% CI
Cronbach’s alpha (α)	0.813	-
Sensitivity	95.56%	85.17-98.77
Specificity	88.89%	76.50-95.16
Positive predictive value	89.58%	77.83-95.47
Negative predictive value	95.24%	84.21-98.68
Diagnostic accuracy	92.22%	84.81-96.18
Cohen’s kappa (κ)	0.844	0.64-1.05

The analysis of agreement also confirmed that there was an excellent concordance between the questionnaire and biomedical diagnosis, supporting that the instrument is a reliable screening tool.

## Discussion

This study demonstrated a close relationship between MetS and MSD, consistent with the contemporary understanding of metabolic disease as a state of metabolic dysregulation. The absence of significant differences in age and sex between individuals with and without MetS suggests that, within the mid-life age group, adiposity and lifestyle-related factors may play a more critical role in determining metabolic risk than chronological age or sex. These findings are consistent with large population-based studies in India, indicating that the prevalence of MetS increases after early adulthood and stabilises with the progression of obesity and insulin resistance [12,13].

From an Ayurvedic perspective, this life stage corresponds to *Madhyama Vaya* (middle age), during which metabolic efficiency declines and susceptibility to *Meda Dhatu *(fat tissue) dysfunction increases due to *Agnimandya* (reduced metabolic efficiency) and *Srotorodha* (obstruction of metabolic pathways) [14]. The comparable metabolic risk across sexes further highlights the predominance of modifiable lifestyle factors, such as excessive caloric intake, physical inactivity, and central adiposity, over inherent biological differences.

Anthropometric findings demonstrated significantly higher BMI and waist circumference among participants with MetS, reinforcing the established role of visceral adiposity in metabolic pathology. Visceral adipose tissue functions as an active endocrine organ contributing to insulin resistance, endothelial dysfunction, and systemic inflammation through dysregulated adipokine secretion [15]. These findings are consistent with international and Indian clinical guidelines that identify waist circumference as a stronger predictor of metabolic risk than general adiposity [16]. In Ayurvedic physiology, this pattern corresponds to *Meda Vriddhi *(excess fat accumulation) associated with *Ama-Meda* (metabolic toxins associated with fat tissue) accumulation, leading to *Srotodushti *(channel dysfunction) and progression to *Sthoulya *(obesity), *Prameha *(metabolic disorders), and *Hridroga *(cardiovascular disease) [17]. Clinical features such as abdominal enlargement, heaviness, and excessive perspiration observed in individuals with higher MSD scores further support the construct validity of MSD as a functional correlate of metabolic risk [18,19].

Hemodynamic assessment revealed significantly elevated systolic and diastolic blood pressure among participants with MetS. These findings align with established mechanisms of obesity-related hypertension, including endothelial dysfunction, sympathetic nervous system activation, and renin-angiotensin-aldosterone system involvement [20]. From an Ayurvedic standpoint, these changes correspond to *Meda-Kapha Sanga *(obstruction due to fat and biofluid accumulation) leading to *Vyana Vata Avarodha *(functional obstruction of circulatory dynamics), resulting in impaired circulatory regulation and sustained hypertension. The sequence of *Meda Vriddhi -* *Srotorodha -* *Vyana Vata Dushti *(circulatory dysregulation) *-* *Uchcha Rakta Chapa* (hypertension) parallels biomedical explanations of obesity-induced hypertension [21].

Biochemical findings showed elevated serum triglycerides and fasting blood glucose, along with reduced HDL cholesterol, consistent with the atherogenic dyslipidemia characteristic of MetS [22]. Ayurveda attributes such abnormalities to *Meda Dhatu Agnimandya* (diminished metabolic activity of fat tissue), resulting in *Ama-Meda* accumulation and obstruction of metabolic and circulatory channels [23]. The similarity between classical descriptions of *Prameha Purvarupa* (prodromal symptoms of metabolic disorders) and contemporary manifestations of hyperglycemia and dyslipidemia suggests that MSD may reflect early functional manifestations of metabolic imbalance [24,25].

Integratively, the biomedical progression from visceral adiposity to insulin resistance, dyslipidemia, and hyperglycemia can be compared with the Ayurvedic cascade of *Agnimandya *- *Ama-Meda Nirmana* (formation of toxic fat metabolites) - *Srotorodha *- *Sthoulya *- *Prameha *[26,27]. The high diagnostic agreement observed between MSD questionnaire classification and MetS criteria suggests that MSD may represent a clinically relevant functional correlate of metabolic disturbances; however, this association reflects cross-sectional concordance and does not establish causality or independent predictive validity.

Multivariable analysis identified obesity as the most significant independent predictor of MetS after adjustment for sex and MSD, reflecting its central role in metabolic disease progression. Although MSD showed a strong unadjusted association with MetS, its lack of independent significance in adjusted analysis indicates possible confounding, particularly by obesity, and limits conclusions regarding its independent predictive role. These findings further indicate that the observed diagnostic performance reflects association and agreement rather than independent predictive capability. The observed diagnostic performance of the MSD questionnaire, including high sensitivity, specificity, and agreement with MetS diagnosis, suggests its potential as a supportive non-invasive adjunct screening tool rather than a standalone diagnostic or predictive instrument; however, its independent clinical utility requires further validation in larger longitudinal studies.

This study demonstrates several methodological strengths, including the use of a predefined and psychometrically validated questionnaire, integration of both subjective symptom-based assessment and objective biochemical and anthropometric measurements, and comprehensive evaluation of diagnostic performance indices along with agreement analysis.

Limitations and future recommendations

This study has several limitations that should be considered when interpreting the findings. The cross-sectional design precludes causal inference, and the single-centre setting with a relatively small sample size may limit generalisability. The use of quota sampling with predefined equal representation of MSD-positive and MSD-negative groups may have introduced selection bias and spectrum bias, potentially inflating diagnostic accuracy estimates and limiting external validity. Additionally, the symptom-based nature of the MSD questionnaire involves partial self-reporting, although this is supported by strong psychometric reliability. The limited sample size may also contribute to instability in regression estimates, as reflected in wide CIs.

Although MSD demonstrated a strong unadjusted association with MetS, its lack of independent significance in multivariable analysis suggests potential confounding, particularly by obesity, and warrants cautious interpretation. The diagnostic cutoff for the MSD questionnaire was adopted from prior validation studies and was not derived using ROC curve analysis in the present study, which may influence the precision of the threshold. Furthermore, exclusion of participants with established metabolic disorders may limit the applicability of findings to broader populations.

These limitations, particularly those related to sampling strategy and study design, should be considered when interpreting the reported diagnostic performance and generalisability of the findings.

Future studies should be multicentric and longitudinal to evaluate the predictive role of MSD in the development of MetS and related cardiometabolic conditions. Such studies may also help clarify the temporal relationship between early functional metabolic alterations and subsequent biochemical and structural changes. Incorporating random sampling methods and ROC-based cutoff validation would enhance methodological robustness and external validity. Further research exploring the responsiveness of the MSD questionnaire to lifestyle or metabolic interventions may strengthen its role as an adjunct tool in preventive and integrative healthcare models.

## Conclusions

The findings of this study demonstrate a significant association between MSD and MetS, indicating concordance with established biomedical characteristics of metabolic dysfunction. The results suggest that MSD may reflect underlying disturbances in lipid and glucose metabolism consistent with known cardiometabolic risk factors, and that the MSD questionnaire provides a non-invasive and practical adjunct approach for assessing functional metabolic alterations, with high diagnostic agreement observed in this study. However, given the cross-sectional design, potential selection bias, and the lack of independent association in multivariable analysis, these findings should be interpreted with caution as they do not establish causal relationships or independent predictive validity. Accordingly, the MSD questionnaire should not be considered a standalone diagnostic or predictive instrument, but rather a complementary tool within an integrative metabolic risk assessment framework. The study supports the potential role of integrating Ayurvedic functional assessment with conventional metabolic evaluation; however, the findings remain preliminary and require validation through larger, multicentric, and longitudinal studies to establish clinical utility and generalisability.

## Appendices

Appendix A

**Table 7:** MSD assessment questionnaire This questionnaire was developed by the authors. MSD: *Medovaha Srotas Dushti*

S. No.	Sanskrit Term	English Question	Yes (Score - 1)	No (Score - 0)
1	*Jatilbhava kesesu *(matting of hairs)	Does your hair feel sticky or get tangled easily, making it hard to comb and requiring washing more than twice a week?		
2	*Madhuryam asyasya *(sweetness in the mouth)	Do you often experience a sweet taste or sticky feeling in your mouth, even after brushing your teeth?		
3	*Karapadayoh dahah *(burning sensation in palms and soles)	Do you experience a burning sensation in your palms and soles?		
4	*Karapadayoh suptata* (numbness in hands and feet)	Do you often feel numbness or loss of sensation in your hands and feet?		
5	*Mukha talu kantha sosah* (dryness in mouth, palate, and throat)	Do you often feel dryness in your mouth, palate, or throat even after drinking adequate fluids?		
6	*Pipasa *(thirst)	Do you feel intense or unusual thirst throughout the day, even after drinking normal amounts of fluids?		
7	*Alasyam *(lassitude)	Do you often feel lethargic or lazy even after resting or sleeping?		
8	*Malam kaye *(dirt in body)	Do you feel that your body remains dirty or sticky with sweat even after taking a bath?		
9	*Kayacchidresu upadeham* (smearing in body orifices)	Have you noticed unusual discharge or secretion from body orifices such as the ear, armpits, teeth, or nostrils, causing discomfort or irritation?		
10	*Satpadapipilikabhih sarira mutrabisharanam *(sensation of insects crawling on the body or attraction of ants/flies to urine)	Do you feel sensations similar to insects crawling on or under your skin, or notice flies gathering when you urinate?		
11	*Mutre ca mutrabodhanam* (abnormal changes in urine characteristics)	Have you noticed unusual changes in the color, appearance, or smell of your urine?		
12	*Visram sariragandham* (fleshy smell from the body)	Do others notice a strong or unpleasant odor coming from your body?		
13	*Nidram tandram sarvakalam iti* (frequent sleep)	Do you frequently feel excessively sleepy or tired even while trying to remain active?		
